# Mouse Models of Diet-Induced Nonalcoholic Steatohepatitis Reproduce the Heterogeneity of the Human Disease

**DOI:** 10.1371/journal.pone.0127991

**Published:** 2015-05-27

**Authors:** Mariana Verdelho Machado, Gregory Alexander Michelotti, Guanhua Xie, Thiago Pereira de Almeida, Jerome Boursier, Brittany Bohnic, Cynthia D. Guy, Anna Mae Diehl

**Affiliations:** 1 Division of Gastroenterology, Department of Medicine, Duke University Medical Center, Durham, NC 27710, United States of America; 2 Gastroenterology Department, Hospital de Santa Maria, CHLN, Lisbon, Portugal; 3 Division of Endocrinology, Duke University Medical Center, Durham, NC 27710, United States of America; 4 Division of Pathology, Duke University Medical Center, Durham, NC 27710, United States of America; Institute of Medical Research A Lanari-IDIM, University of Buenos Aires-National Council of Scientific and Technological Research (CONICET), ARGENTINA

## Abstract

**Background and aims:**

Non-alcoholic steatohepatitis (NASH), the potentially progressive form of nonalcoholic fatty liver disease (NAFLD), is the pandemic liver disease of our time. Although there are several animal models of NASH, consensus regarding the optimal model is lacking. We aimed to compare features of NASH in the two most widely-used mouse models: methionine-choline deficient (MCD) diet and Western diet.

**Methods:**

Mice were fed standard chow, MCD diet for 8 weeks, or Western diet (45% energy from fat, predominantly saturated fat, with 0.2% cholesterol, plus drinking water supplemented with fructose and glucose) for 16 weeks. Liver pathology and metabolic profile were compared.

**Results:**

The metabolic profile associated with human NASH was better mimicked by Western diet. Although hepatic steatosis (i.e., triglyceride accumulation) was also more severe, liver non-esterified fatty acid content was lower than in the MCD diet group. NASH was also less severe and less reproducible in the Western diet model, as evidenced by less liver cell death/apoptosis, inflammation, ductular reaction, and fibrosis. Various mechanisms implicated in human NASH pathogenesis/progression were also less robust in the Western diet model, including oxidative stress, ER stress, autophagy deregulation, and hedgehog pathway activation.

**Conclusion:**

Feeding mice a Western diet models metabolic perturbations that are common in humans with mild NASH, whereas administration of a MCD diet better models the pathobiological mechanisms that cause human NAFLD to progress to advanced NASH.

## Introduction

Nonalcoholic fatty liver disease (NAFLD) is the most prevalent liver disease in Western society [1]. It is estimated that one billion subjects have NAFLD worldwide [2]. Although the majority of patients will have a benign evolution, up to 25% develop potentially progressive liver damage, dubbed nonalcoholic steatohepatitis (NASH). NASH is characterized by liver cell injury/death, inflammation, and increased risk for liver fibrosis and carcinogenesis [3]. NAFLD/NASH is the third leading indication for liver transplantation in the US, and the second cause for hepatocellular carcinoma-related liver transplantation [4].

Though much has been learned about NAFLD since its first descriptions thirty years ago, there are still huge gaps in knowledge regarding its pathogenesis, prognosis, prevention, and treatment. Studying human NAFLD is hampered by the fact that it encompasses a spectrum of conditions difficult to differentiate non-invasively. Also, NAFLD is a very slowly progressive disease, which hinders prospective observational studies. Given these challenges, animal models that mimic human pathology are a necessity. The perfect animal model would develop NAFLD in the context of key risk factors for the human condition (i.e., obesity and the metabolic syndrome, MS), eventually manifest all histological features of NASH, progress to advanced liver fibrosis, and be susceptible to hepatocellular carcinoma. Importantly, the ideal NASH model should require little time and expense to develop/maintain, and be highly reproducible. There are several diet-induced models of NAFLD/NASH in small animals. Mice are generally preferred due to their short lifespan and the ease of genetically manipulating putative pathogenic/protective pathways.

The most widely used diet to induce NAFLD/NASH is the methionine-choline deficient (MCD) diet. Standard MCD diet also has a high content of sucrose (40% of energy) and is moderately enriched with fat (10–20%). It is a very reproducible model, consistently inducing a phenotype of severe NASH after 8 weeks of administration [5]. The MCD diet has been criticized, however, because it causes weight loss and does not induce features of the MS, an important risk factor for NAFLD [6]. High-fat diet is another highly studied approach to develop NAFLD. The heterogeneity of such diets makes it difficult to compare studies from different research groups. However, standard high fat diets generally do not induce significant NASH (i.e., liver cell death, inflammation, or fibrosis) even when fed for more than 28 weeks, despite reproducibly provoking obesity, the MS, and hepatic steatosis [7]. More recently, a modified high-fat diet has been used to model of NAFLD/NASH. This diet is enriched in saturated fatty acids and supplemented with cholesterol as well as high-fructose corn syrup equivalents, mimicking the fast food style that characterizes the “Western diet” [8]. The Western diet has the advantage of inducing obesity and the MS in mice, although requiring long-term administration.

In this study, we performed a head-to-head comparison of the MCD and Western diets after controlling for multiple variables that impact metabolism and NAFLD pathogenesis: mouse genetic background, age, gender, and inter-animal facility-related differences in light-dark cycling and indigenous microbial flora. The results identify important diet-induced differences that may explain why the two diets model different stages of NAFLD pathogenesis.

## Methods

### Animal Studies

Male wild-type (WT) mice C57Bl/6 were obtained from Jackson Laboratory and fed either chow diet (Picolab Rodent diet 20, #5053; n = 13 mice); MCD diet (MP Biomedicals, #960439; n = 15 mice) for 8 weeks, or Western diet (TD.120330 22% HVO + 0.2% cholesterol diet, Teklad Research, supplemented with high-corn fructose syrup-equivalents in the drinking water, that is 42 g/L glucose and fructose, 55% and 45% respectively, w/w; n = 8 mice) for 16 weeks. The characteristics of the diets are summarized in S1 and S2 Tables. Interventions were started at 12 weeks of age for MCD diet and 4 weeks for Western diet, in order to complete the protocol at 20 weeks of age in all mice. Animal care and procedures were approved by the Duke University Institutional Animal Care and fulfilled National Institutes for Health and Duke University IACUC requirements for humane animal care.

### Human Samples

Liver biopsies from liver transplant donors (n = 5) and adult NASH patients with mild fibrosis (F0-1) (n = 5) or severe fibrosis (F3-4) (n = 5) were randomly selected from Duke University Health System NAFLD Clinical Database and Biorepository. Patients gave informed consent at time of recruitment. Prior to this analysis patient records were anonymized and de-identified. Studies were approved by the Duke IRB and conducted in accordance with National Institutes of Health and institutional guidelines for human subject research.

### Histopathological analysis

Formalin-fixed, paraffin-embedded liver sections were stained with H&E and evaluated for severity of NAFLD, by a trained pathologist, according to criteria described by Brunt *et al*. [9]. Immunohistochemistry was done, as previously described, with the antibodies specified in S3 Table [10]. Liver fibrosis was assessed by Picrosirius red (#365548, Sigma) staining [10]. TUNEL staining (11684817910, Roche) was performed according to the manufacturer’s suggestions.

### Serum and tissue analysis

Liver enzymes were assayed by the Veterinary Diagnostic Laboratory, Division of Laboratory Animal Resources, Duke University Medical Center. Insulin was measured with Ultrasensitive Mouse Insulin ELISA kit (Crystal Chem Inc: #90080); lipids were measured in the serum and liver with Triglyceride Colorimetric Assay kit (Cayman Chemical Company: #10010303), Free Fatty Acid Quantification Kit, (Abcam, ab65341) and Cholesterol Quantification kit (Abcam, ab65359); serum leptin and adiponectin were determined with Abcam mouse ELISA kits, ab100718 and ab108785, respectively. Liver hydroxyproline content was quantified as previously described [11].

### Molecular Studies

#### mRNA quantification by Real-time Reverse Transcription-PCR (RT-PCR)

Total RNA was extracted from livers using TRIzol (Invitrogen). RNA was reverse transcribed to cDNA templates using random primer and Super Script RNAse H-Reverse Transcriptase (Invitrogen) and amplified. Semiquantitative qRT-PCR was performed using iQ-SYBR Green Supermix (Bio-Rad) and StepOne Plus Real-Time PCR Platform (ABI/Life Technologies), as previously described [12]. For primers, see S4 Table.

#### Western Blotting

Total proteins were extracted from whole liver using RIPA buffer (Sigma). Proteins were separated by electrophoresis on 4%-20% Criterion gels (BioRad), transblotted into polyvinylidene-difluoride membranes, and incubated with primary antibodies listed in S5 Table.

### Statistics

Results were expressed as mean±SEM. Significance was established using Kruskal-Wallis and Mann-Whitney tests, with significance p<0.05, corrected for multiple comparisons.

## Results

### Western diet mimics metabolic profile associated with human NASH

**Fig 1:**
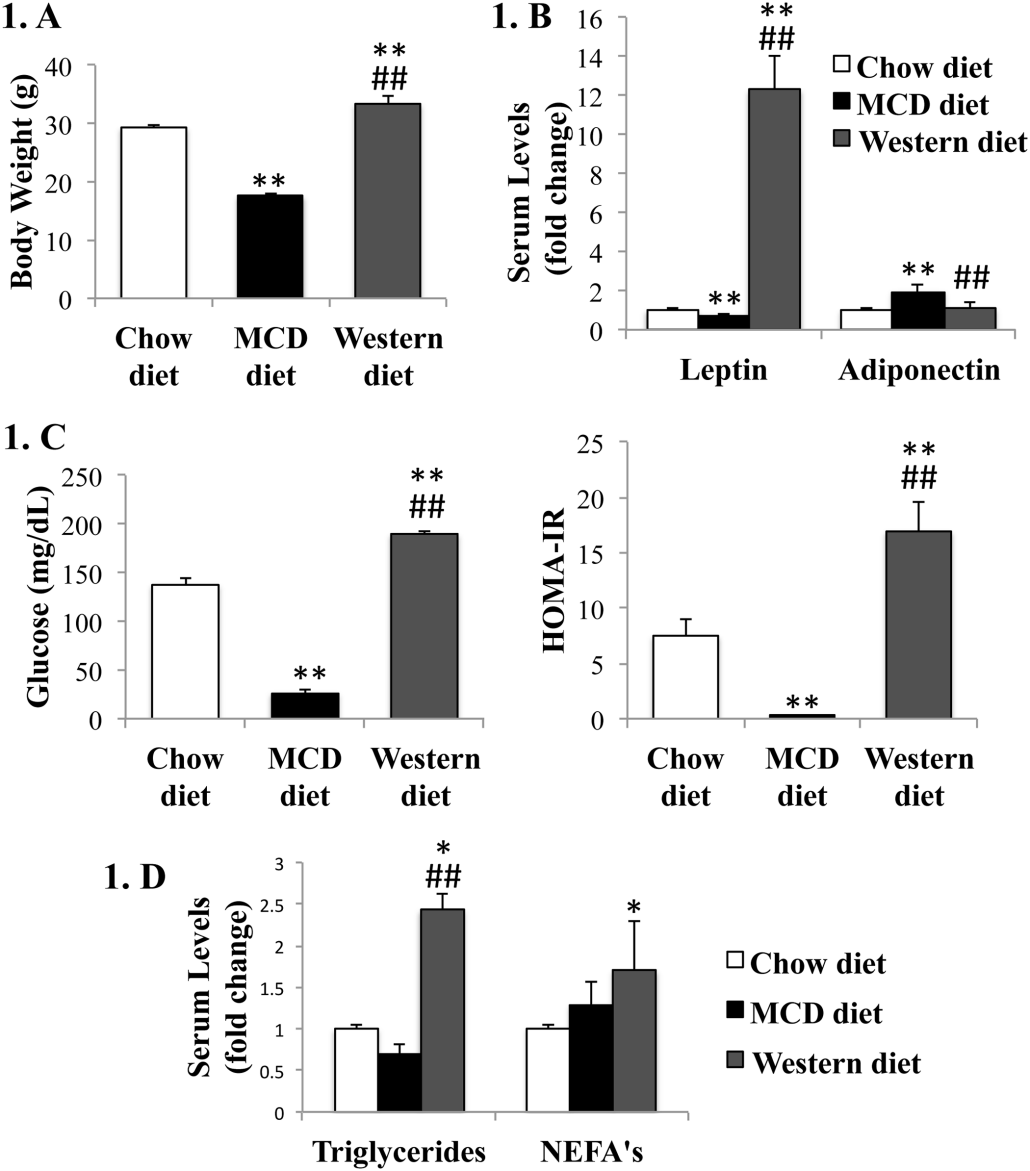
Effects of MCD diet and Western diet on metabolic profile. WT mice were fed chow diet, MCD diet for 8 weeks, or Western diet for 16 weeks, and sacrificed at 20 weeks of age. (A). Body weights; (B). Adipokines (leptin and adiponectin); (C). Fasting serum glucose and HOMA-IR index; (D). Serum triglycerides and non-esterified fatty acids (NEFA’s). Mean±SEM results are graphed. *<0.05 and **<0.005, control *versus* experimental diet; #<0.05 and ##<0.005, MCD *versus* Western diet.

Twenty-week old wild-type mice that had been fed chow diet, MCD diet for 8 weeks, or Western diet for 16 weeks were studied. MCD diet-fed animals weighed 40% less, and Western diet-fed animals 14% more, than age- and gender-matched chow-fed mice (Fig 1A). Weight loss in the MCD diet-fed group was more pronounced in the beginning of the diet. On average, mice lost 10% body mass/week during the first 2 weeks of MCD diet, 5% in week 3, and 2.5%/week thereafter. MCD diet-related weight loss has been attributed to hypermetabolism [13] caused by increased sympathetic nervous system outflow to adipose tissue [14], with resultant increased mitochondrial uncoupling leading to less efficient energy extraction from nutrients [15]. We observed increased uncoupling protein gene expression in the livers of MCD-fed mice (S1 Fig).

As expected, the Western diet promoted obesity, though with modest weight gain as compared to controls. This excessive weight gain was noteworthy, however, because the Western diet-fed group consumed only half as much solid food as controls fed isocaloric standard chow, as expected given appetite suppressing effect of lipids [16]. On the other hand, the Western diet group was permitted *ad libitum* access to drinking water supplemented with high fructose corn syrup, which associates with decreased satiety and obesity [17]. Concordant with the differences in weight, MCD-diet fed mice were hypoleptinemic and hyperadiponectinemic, whereas Western-diet fed mice were hyperleptinemic relative to controls (Fig 1B). This is concordant with the literature, since increase in adiponectin has been described in the MCD diet, however MCD diet also seems to associate with hepatic adiponectin resistance [18]. Western diet mimicked the MS associated with human NAFLD/NASH, with hyperglycemia/insulin resistance (IR) as well as dyslipidemia. In contrast, MCD-diet fed mice did not exhibit hyperlipidemia or IR and became severely hypoglycemic with fasting (Fig 1D and 1C). Of note, although mice fed the MCD diet develop peripheral insulin sensitivity, it is known that MCD diet associates with liver insulin resistance, which may mimic the liver effects of metabolic syndrome-phenotype associated with human NAFLD [19,20].

### MCD diet induces less hepatic steatosis, but more liver injury, than Western diet

**Fig 2:**
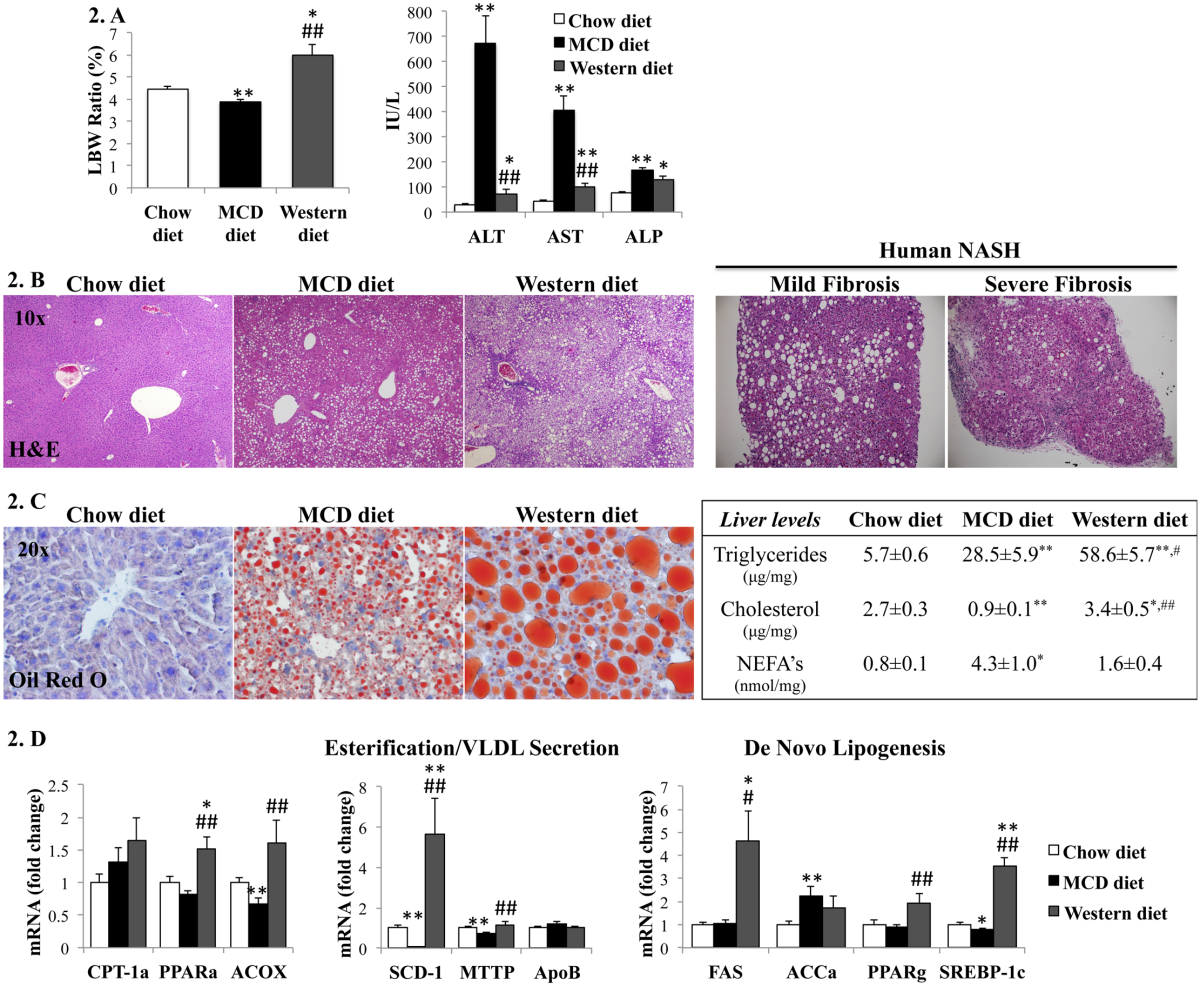
Effects of MCD diet and Western diet on hepatic steatosis and liver enzymes. (A). Liver to body weight (LBW) ratio, serum aminotransferases (ALT, AST) and alkaline phosphatase (ALP) from mice fed chow, MCD or Western diets. (B). H&E staining of representative liver sections from mice (left panels) and NASH patients with mild or severe fibrosis (right panels). (C). Oil-red O staining of representative liver sections from mice (left panels) and liver lipid levels (right). (D). qRT-PCR analysis of liver genes encoding lipid metabolic enzymes. Results normalized to chow-diet fed mice and graphed as mean±SEM. *<0.05 and **<0.005, control *versus* experimental diet; #<0.05 and ##<0.005, MCD *versus* Western diet.

Although both diets induced liver steatosis, only Western diet-fed mice developed hepatomegaly (i.e., increased liver-to-body weight (LBW) ratio) (Fig 2A). LBW in MCD-diet fed mice was actually lower than controls, suggesting that MCD diets led to loss of liver mass. Indeed, serum liver enzymes, as well as histologic liver inflammation and the NAS score, were all significantly greater in the MCD diet-fed mice than the Western diet-fed mice, supporting the concept that the MCD diet provoked worse liver damage (Fig 2C). Although neither diet induced clear pathological findings of hepatocellular ballooning, a hallmark lesion in human-NASH, the MCD diet better mimicked the other pathological findings typical of severe human NASH (Fig 2B), namely lobular/periportal inflammation and perivenular/perisinusoidal fibrosis.

### Hepatic lipid composition and mechanisms of fat accumulation differ in Western diet and MCD diet models

Western diet induced more impressive hepatic steatosis than MCD diet, as assessed by oil-red staining and liver triglyceride concentration. The Western diet supplemented cholesterol and hence increased liver cholesterol content, whereas liver cholesterol content in MCD diet-fed mice was actually lower than chow-fed controls. Interestingly, liver non-esterified fatty acids (NEFA’s), which are believed to be the more active and injury-inducing lipids [21], were higher in the MCD-diet group (Fig 2C). The mechanisms driving liver fat accumulation also seemed to differ in the two models. Western diet induced genes involved in *de novo* lipogenesis, whereas MCD diet caused down-regulation of genes for fatty acid esterification and very low-density lipoprotein (VLDL) secretion (Fig 2D).

### Fibrosis and ductular response worse and more consistent in MCD diet model

**Fig 3:**
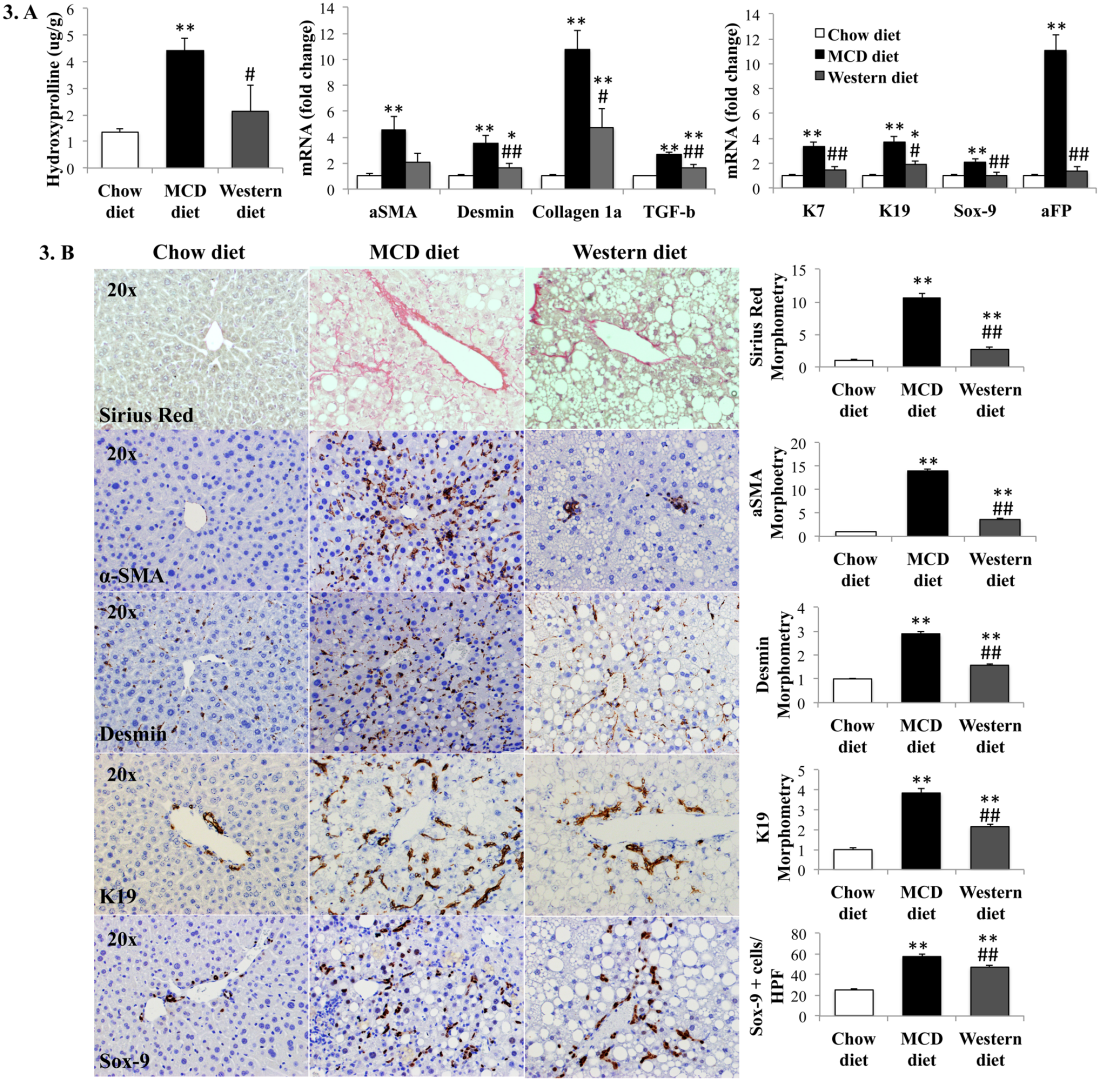
Effects of MCD diet and Western diet on liver fibrosis and ductular reaction. (A). Hepatic hydroxyl-proline content and qRT-PCR analysis of liver fibrosis and progenitor marker genes; (B). Liver sections from representative mice stained for markers of the fibroductular reaction (Sirius red, α-SMA, desmin, K19 and Sox-9) (left panels) and respective morphometry (right). Results normalized to chow-fed mice and graphed as mean±SEM. *<0.05 and **<0.005, control *versus* experimental diet; #<0.05 and ##<0.005, MCD *versus* Western diet.

MCD diet was significantly more fibrogenic than Western diet, as assessed by hydroxyproline assay, Sirius red staining, gene expression analysis and immunohistochemistry for stellate cell activation markers (Fig 3). Of note, animal-to-animal variability in the severity of liver fibrosis was greater in the Western diet than in the MCD diet group. Ductular response intensity (assessed by qRT-PCR analysis and immunohistochemistry of progenitor marker genes) generally paralleled the severity of the fibrogenic response, both being significantly greater in the MCD diet than the Western diet model (Fig 3). Most notably, αSMA and K19 immunostaining in the MCD diet-fed mice was quantitatively and qualitatively similar to that of NASH patients with severe fibrosis (S2 Fig).

### Worse hepatic apoptosis and inflammation in MCD diet model

**Fig 4:**
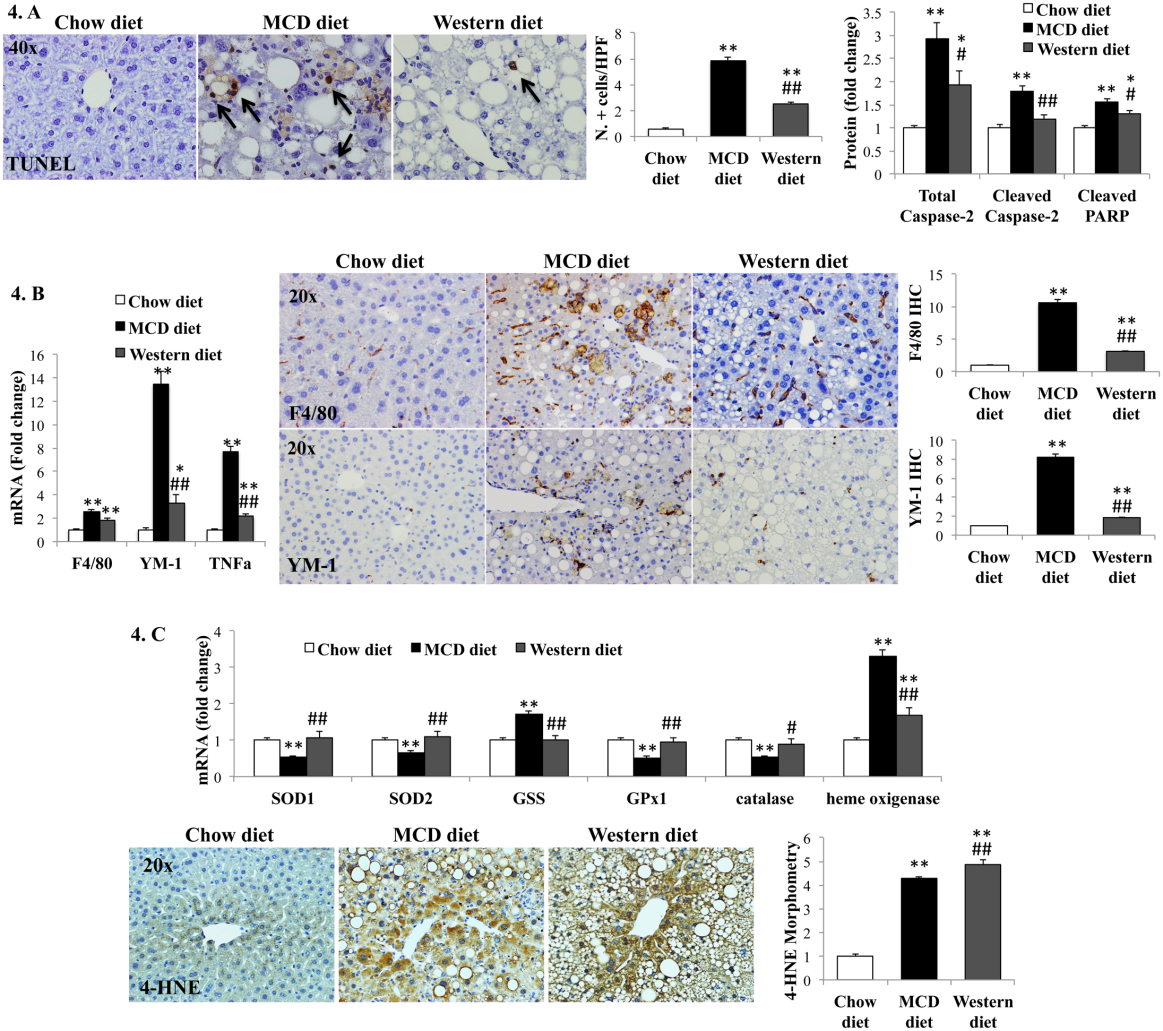
Effects of MCD diet and Western diet on liver cell death, inflammation and oxidative stress. (A). TUNEL assay and Western blot for caspase-2 and cleaved PARP; (B). qRT-PCR of macrophage markers (F4/80 and YM-1) and TNF-α (left). Immunohistochemistry for F4/80 and YM-1: Representative photos and morphometry (right). (C). qRT-PCR analysis of anti-oxidant enzymes (top) and immunohistochemistry plus morphometry for 4-hydroxynonenal (4-HNE) in representative mice (lower). Results normalized to chow-diet fed mice and graphed as mean±SEM. *<0.05 and **<0.005, control *versus* experimental diet; ##<0.005, MCD *versus* Western diet.

Caspase-2 is an important initiator of lipotoxicity-related hepatocyte apoptosis in animal models of NASH, and its expression correlates with NASH severity in humans [22]. MCD diet induced more caspase-2 expression than Western diet (Fig 4A and S3 Fig). Cleaved PARP, a marker of active apoptosis, and numbers of TUNEL-positive cells (another indicator of cell death) were both significantly greater in MCD diet-fed mice than Western diet-fed mice (Fig 4A and S3 Fig). MCD diet induced more liver inflammation than Western diet, as evidenced by higher liver mRNA and protein levels of TNFα, F4/80 and YM-1, markers of classical and alternative macrophage activation, respectively (Fig 4B). Infiltration by other immune cells, namely lymphocytes, monocytes and neutrophils, though less marked than macrophage accumulation, was also higher in MCD diet-fed mice compared to Western diet-fed animals, as assessed by qRT-PCR analysis of cell-type specific markers (S4 Fig).

### MCD diet models mechanisms implicated in human NASH pathogenesis/progression

**Fig 5:**
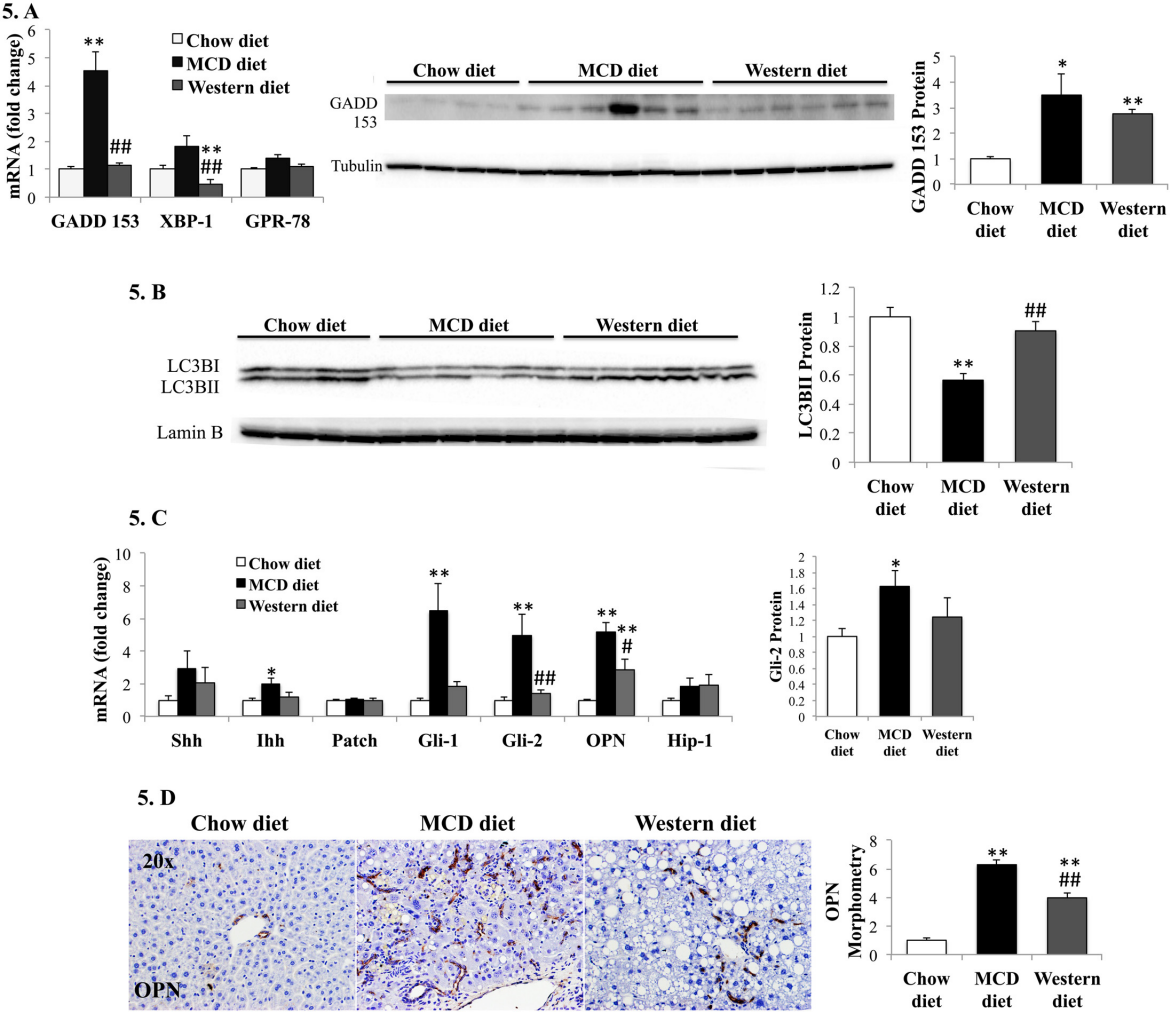
Effects of MCD diet and Western diet on liver cell stress and Hedgehog pathway. (A). qRT-PCR analysis of relevant ER stress-related genes (left panel) and western blot analysis of GADD-153 (right panel); (C). Western blot analysis of the autophagy marker, LC3BII. (C). qRT-PCR analysis of Hedgehog ligands (Shh, Ihh), receptor (Patch), Hedgehog-regulated transcription factors (Gli1, Gli2), and Hedgehog-target genes (OPN, Hip-1) (left); Western blot analysis of Gli-2 (right). (D). Immunohistochemistry for osteopontin: sections from representative mice and morphometry. Results graphed as mean±SEM. *<0.05 and **<0.005, control *versus* experimental diet; #<0.05 and ##<0.005, MCD *versus* Western diet.

Oxidative stress plays a major role in the pathogenesis of human NASH [23,24]. To limit liver injury, hydroxyl radical (^**.**^OH), nitric oxide radical (NO^**.**^), and superoxide anion (O_2_^**.**-^) must be neutralized by antioxidant enzymes (superoxide dismutases) which convert O_2_^**.**-^ into H_2_O_2._ The latter is further detoxified by glutathione peroxidase (GPx) or catalase. Similar to humans with severe NASH [25,26], mice fed MCD diet demonstrated decreased expression of antioxidant enzymes (Fig 4C). MCD diet is also known to deplete S-adenosyl methionine, a methyl donor important for glutathione synthesis and anti-oxidant defense [27]. This suggests that MCD diet imposes greater oxidative stress than Western diet. Consistent with this, gene expression of heme oxygenase (a marker of oxidative stress) [28], was higher in MCD diet-fed mice than Western diet-fed mice. Also, MCD diet- and Western diet- groups had comparable 4-hydroxynonenal accumulation despite the fact that MCD diet-fed mice had lower hepatic lipid content and thus, less substrate for lipoperoxide production (Fig 4C). Endoplasmic reticulum (ER) stress and the consequent unfolded protein response (UPR) are other key factors in the pathogenesis of human NAFLD/NASH [29–31]. MCD-diet provoked greater induction of UPR mediators, transcription factor X-box binding protein-1, XBP-1, and chaperone glucose regulated protein-78, GPR-78, than Western diet. It also stimulated greater expression of growth-arrest and DNA damage-inducible gene-153, GADD153, an important mediator of ER stress-eliciting apoptosis response (Fig 5A). Deregulated autophagy occurs in IR and obesity [32], and has also been implicated in NASH pathogenesis. Autophagy and steatosis severity are inversely correlated [33]. MCD diet-fed mice demonstrated evidence for decreased autophagy based on reduced hepatic accumulation of the active conjugate form of microtubule-associated protein 1 light chain 3, LC3-II (Fig 5B). Thus, three key cell stress-related mechanisms that have been implicated in human NASH pathogenesis (oxidative stress, ER stress, and autophagocytic stress) are significantly more active in the MCD diet model than in the Western diet model.

Finally, Hedgehog pathway activation is pivotal in NASH progression, driving profibrogenic responses in animal models and correlating with fibrosis in human NASH [11,22,34–36]. The Hedgehog pathway was more activated in MCD diet-fed mice than Western diet-fed mice, with greater induction of Hedgehog ligands and Hedgehog-target genes (Fig 5C and 5D).

## Discussion

Efficient, inexpensive, reproducible, and relevant animal models are needed to advance understanding of the mechanisms that drive the pathogenesis and progression of NASH in humans. Two diets, MCD diet and Western diet, have been widely used to model human NASH in rodents. Each approach has its advocates, who often condemn the alternative model for failing to faithfully mimic relevant human pathobiology. The present head-to-head comparison of the two models suggests that such controversy is groundless because each diet beautifully models a different sub-type of human NASH.

Human NAFLD is a heterogeneous disease. Although NAFLD is extremely prevalent, only a minor sub-population of individuals with hepatic steatosis demonstrate NASH at any given point in time [2]. Natural history studies suggest that bad liver-related outcomes (i.e., cirrhosis and liver cancer) are more likely to occur in the NASH sub-group, but also clearly indicate that progression is far from inevitable [3]. Indeed, “spontaneous” regression of NASH has been documented to occur in some of the placebo group participants in several prospective NASH treatment trials [37,38]. The variable propensity to improve *versus* progress that exists among individuals with NASH is not well understood. Of note, body mass index/obesity does not seem to be an independent predictor of fibrosis progression or explain the occurrence of rapid fibrosis progression in the 20% of patients that develop progressive fibrosis [3].

Clarifying mechanisms that determine whether or not NASH progresses is a major unmet need because such information would provide the basis for risk stratification and more personalized management. Our results show that the Western diet models the more common, relatively non-progressive subtype of NASH, whereas the MCD diet models the less common, more rapidly progressive/aggressive NASH subtype. Because our study was designed to control for genetic and epigenetic factors that are likely to modulate NASH outcomes in humans (i.e., genetic background, gender, age, and living conditions), diet-sensitive factors that modulate NASH were revealed. The results show that Western diet components are relatively weak NASH agonists, while something about the MCD diet strongly enhances NASH progression. Moreover, the MCD diet-associated factor(s) appear to work by stimulating many of the same mechanisms that promote progressive liver damage in human NASH, including oxidative-, ER-, autophagocytic-stress, and Hedgehog pathway activation.

The Western diet-related factors that promote obesity and insulin-resistance are neither sufficient, nor necessary, for NASH progression in mice. Compared to lean, insulin-sensitive MCD diet-fed mice, which developed severe NASH within 8 weeks, obese, insulin-resistant Western diet-fed mice exhibited only mild NASH even after being exposed to the obesogenic and diabetogenic diet for twice that time. This result has important implications for human NASH. Namely, it underscores the primary importance of non-dietary factors (e.g., genetic background, epigenetic exposures) in driving NASH progression, in obese, insulin-resistant humans. While somewhat surprising, this concept is supported by other independent lines of emerging evidence in both mice and humans. Recent trans-generational studies in mice proved that cirrhosis susceptibility is controlled by epigenetic factors that influence gene methylation [39]. Global differences in hepatic gene methylation patterns have been reported in typical patients with mild *versus* severe NASH [40]. In humans with advanced NASH, genes that encode key methylation-promoting enzymes are differentially hyper-methylated and under-expressed, and the hepatic genome is generally hypo-methylated relative to humans with mild NASH [40]. Given this background, it is noteworthy that MCD diets deplete methyl donors and may promote global hypo-methylation of the hepatic genome [41]. Thus, the aggregate rodent and human data strongly justify further research to identify key epigenetic mechanisms that control liver health and clarify factors that deregulate these processes to promote tissue damage in individuals with the MS. Contrasting model-dependent differences in various epigenetic profiles (e.g., the hepatic methylome) is likely to identify important diagnostic/therapeutic targets in human NASH because the two mouse models demonstrate significant discrepancies in fibrosis severity, and fibrosis severity strongly predicts liver-related outcomes in human NASH [42].

The new data also help to clarify the relative importance of different types of lipids in NASH pathogenesis. Diet-induced obesity and IR were highly efficient tools for generating hepatic triglyceride accumulation. However, triglyceride content *per se* was not predictive of NASH progression, as demonstrated by the fact that liver injury and fibrosis were much more severe in MCD diet-fed mice which had lower hepatic triglyceride content than Western diet-fed mice with non-progressing NASH. High cholesterol diets are known to provoke liver damage in rodents; hypercholesterolemia is certainly associated with human NASH; and cholesterol-lowering interventions have been correlated with milder forms of NASH in patients [43]. Thus, it has been assumed that lowering liver cholesterol content would improve human NASH. However, in the present study, supplementing dietary cholesterol in obese, diabetic mice was not sufficient to trigger NASH progression, although it did significantly increase hepatic cholesterol content. Rather, more severe liver damage occurred in mice that became somewhat depleted of liver cholesterol due to MCD diet administration. In contrast, the hepatic NEFA content and severity of liver damage were strongly correlated. Relative to MCD diets, the obesogenic/diabetogenic Western diet was a relatively weak stimulant for liver NEFA accumulation. This observation has important implications for researchers who wish to investigate the roles of NEFA in NASH pathogenesis. The latter is a particularly timely issue given that polymorphisms of patatin-like phospholipase domain-containing protein-3 (PNPLA3), a gene that controls hepatic NEFA content, is the strongest genetic association with bad NASH outcomes (i.e., cirrhosis and liver cancer) in humans [44].

Our results also suggest how findings about lipids in these diet-induced NASH models might be used to focus analysis of non-dietary models of NASH, such as various genetically-altered mice strains that develop NASH “spontaneously” on chow diets. For example, severe NASH, cirrhosis and liver cancer have been reported to occur in non-obese mice with targeted genetic defects that enhance insulin sensitivity [45], or that disrupt signaling that activates NF-kB (a major pro-inflammatory factor) [46]. Like MCD diet-fed mice, these genetically-altered mice are often dismissed as reagents for studying NASH pathogenesis because they lack factors (e.g., obesity, IR, pro-inflammatory cytokines) that associate with an increased risk for human NASH. It would seem very informative to determine if/how the liver lipid profiles of the various severe NASH strains align. If increased hepatic NEFAs were found to be conserved among murine NASH “progressors”, this would have major diagnostic and therapeutic implications for the management of human NASH.

In conclusion, while the ideal animal model for NAFLD/NASH has yet to be discovered, the currently available MCD and Western dietary models provide complementary tools to study this major human disease. The MCD diet model has the advantage of being more efficient and reproducible for inducing severe liver damage and progressive fibrosis. MCD diet-fed rodents activate mechanisms that have been implicated in human NASH progression. Thus, this diet approach models the subgroup of NASH patients with histologically advanced NASH, and it is ideal for studying mechanisms driving NASH-related inflammation/fibrosis, as well as strategies to inhibit these processes. In contrast, the Western diet model mimics the vast majority of obese NAFLD/NASH patients. Such individuals typically have IR and MS, but relatively mild liver injury. Hence, the Western diet model should be the first choice for studying how NAFLD/NASH impact systemic metabolic and cardiovascular risk for tissue complications related to type 2 diabetes and atherosclerosis. Because liver injury and fibrosis are relatively mild and slowly progressive in the Western diet model, it also provides an invaluable tool for identifying factors that accelerate NASH progression.

## Supporting Information

S1 Fig Effects of MCD diet and Western diet on liver mitochondrial uncoupling. WT mice were fed chow diet, methionine-choline deficient (MCD) diet for 8 weeks, or Western diet for 16 weeks, and sacrificed at 20 weeks of age. qRT-PCR analysis of liver genes encoding mitochondrial uncoupling proteins. Results were normalized to expression in chow-diet fed mice and graphed as mean±SEM. *<0.05 and **<0.005, control *versus* experimental diet; #<0.05 and ##<0.005, MCD *versus* Western diet.
(TIF)

S2 Fig Fibroductular response in human NASH. Representative photographs from immunohistochemistry for α-SMA and K19 in liver sections from healthy donors (N = 5), adult patients with NASH and mild fibrosis (N = 5) or severe fibrosis (N = 5). Table comparing morphometry for the above staining in murine models and human samples. Results are expressed in fold-change from chow diet (mouse model) or normal human liver (human NASH samples), with average±SEM. All results were statistically different from respective controls (P<0.05).
(TIF)

S3 Fig Effects of MCD diet and Western diet in apoptosis. qRT-PCR analysis and Western blot for caspase-2 and cleaved PARP. Results normalized to chow-diet fed mice and graphed as mean±SEM. *<0.05 and **<0.005, control *versus* experimental diet; ##<0.005, MCD *versus* Western diet.
(TIF)

S4 Fig Effects of MCD diet and Western diet in inflammatory markers. qRT-PCR analysis in whole liver, for: CD3 (pan-T lymphocyte marker), CD20 (pan-B lymphocyte marker), CD115 (marker of blood monocytes) and Ly6G (the granulocyte differentiation antigen 1). Results normalized to chow-diet fed mice and graphed as mean±SEM. *<0.05 and **<0.005, control *versus* experimental diet; ##<0.005, MCD *versus* Western diet.
(TIF)

S1 Table Characteristics of the diets. (DOCX)

S2 Table Composition of the experimental diets. (DOCX)

S3 Table Primaries Antibodies for Immunohistochemistry. (DOCX)

S4 Table RT-PCR primers for analysis. (DOCX)

S5 Table Primary Antibodies for Western Blot. (DOCX)
