# Correction: Mouse Models of Diet-Induced Nonalcoholic Steatohepatitis Reproduce the Heterogeneity of the Human Disease

**DOI:** 10.1371/journal.pone.0132315

**Published:** 2015-06-29

**Authors:** Mariana Verdelho Machado, Gregory Alexander Michelotti, Guanhua Xie, Thiago Pereira de Almeida, Jerome Boursier, Brittany Bohnic, Cynthia D. Guy, Anna Mae Diehl

The fourth author’s name in the Author Byline is incorrect. The correct name is Thiago Almeida Pereira. The correct article citation is: Machado MV, Michelotti GA, Xie G, Pereira TA, Boursier J, Bohnic B, et al. (2015) Mouse Models of Diet-Induced Nonalcoholic Steatohepatitis Reproduce the Heterogeneity of the Human Disease. PLoS ONE 10(5): e0127991. doi:10.1371/journal.pone.0127991
