# Supplementary figures and images for: Mouse Models of Diet-Induced Nonalcoholic Steatohepatitis Reproduce the Heterogeneity of the Human Disease
Source: PLoS One. 2015 May 27;10(5):e0127991. doi: 10.1371/journal.pone.0127991 (PMC4446215; doi:10.1371/journal.pone.0127991)

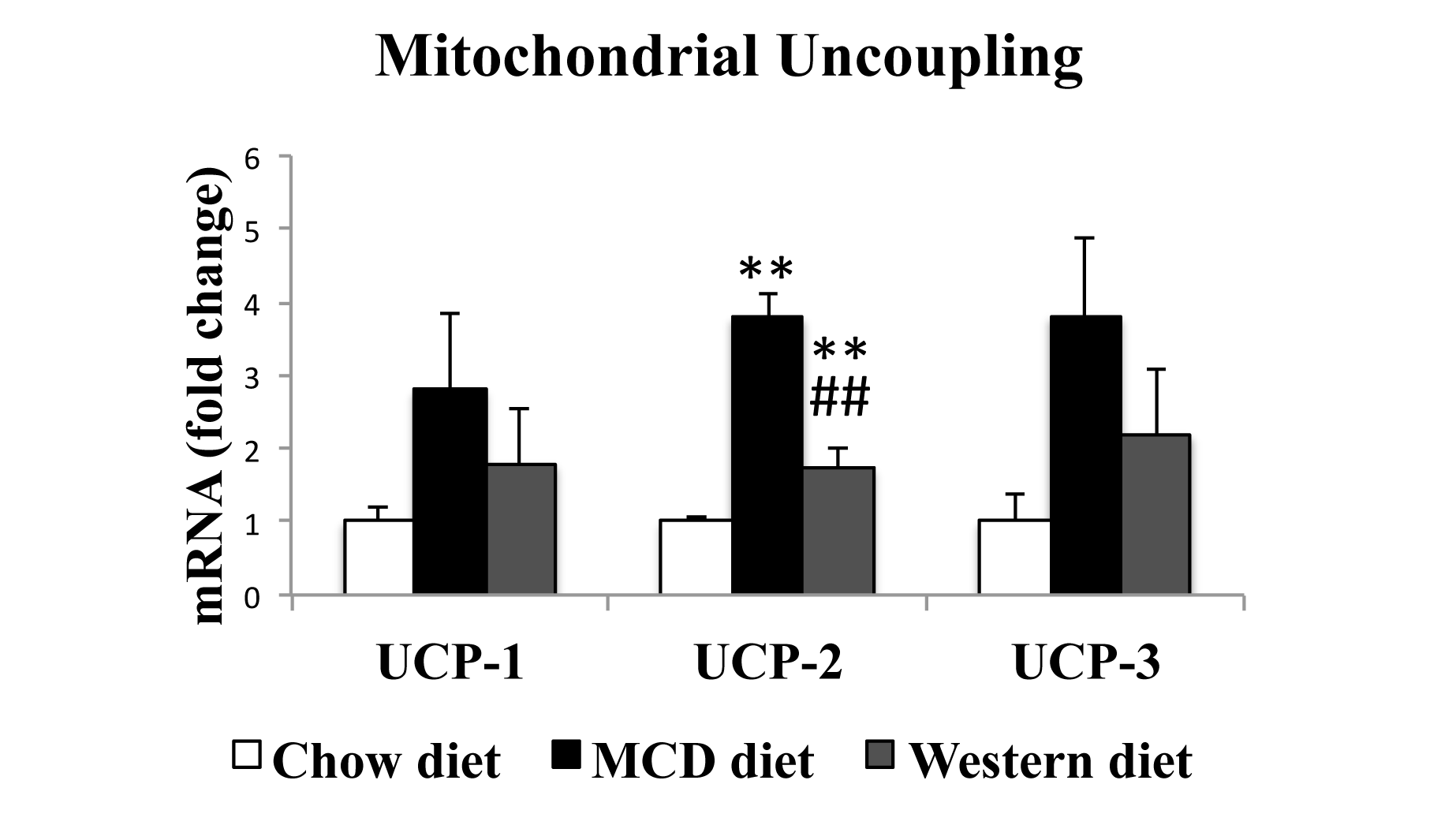

Supplement: S1 Fig — WT mice were fed chow diet, methionine-choline deficient (MCD) diet for 8 weeks, or Western diet for 16 weeks, and sacrificed at 20 weeks of age. qRT-PCR analysis of liver genes encoding mitochondrial uncoupling proteins. Results were normalized to expression in chow-diet fed mice and graphed as mean±SEM. *<0.05 and **<0.005, control versus experimental diet; #<0.05 and ##<0.005, MCD versus Western diet. (TIF) [file pone.0127991.s001.tif]

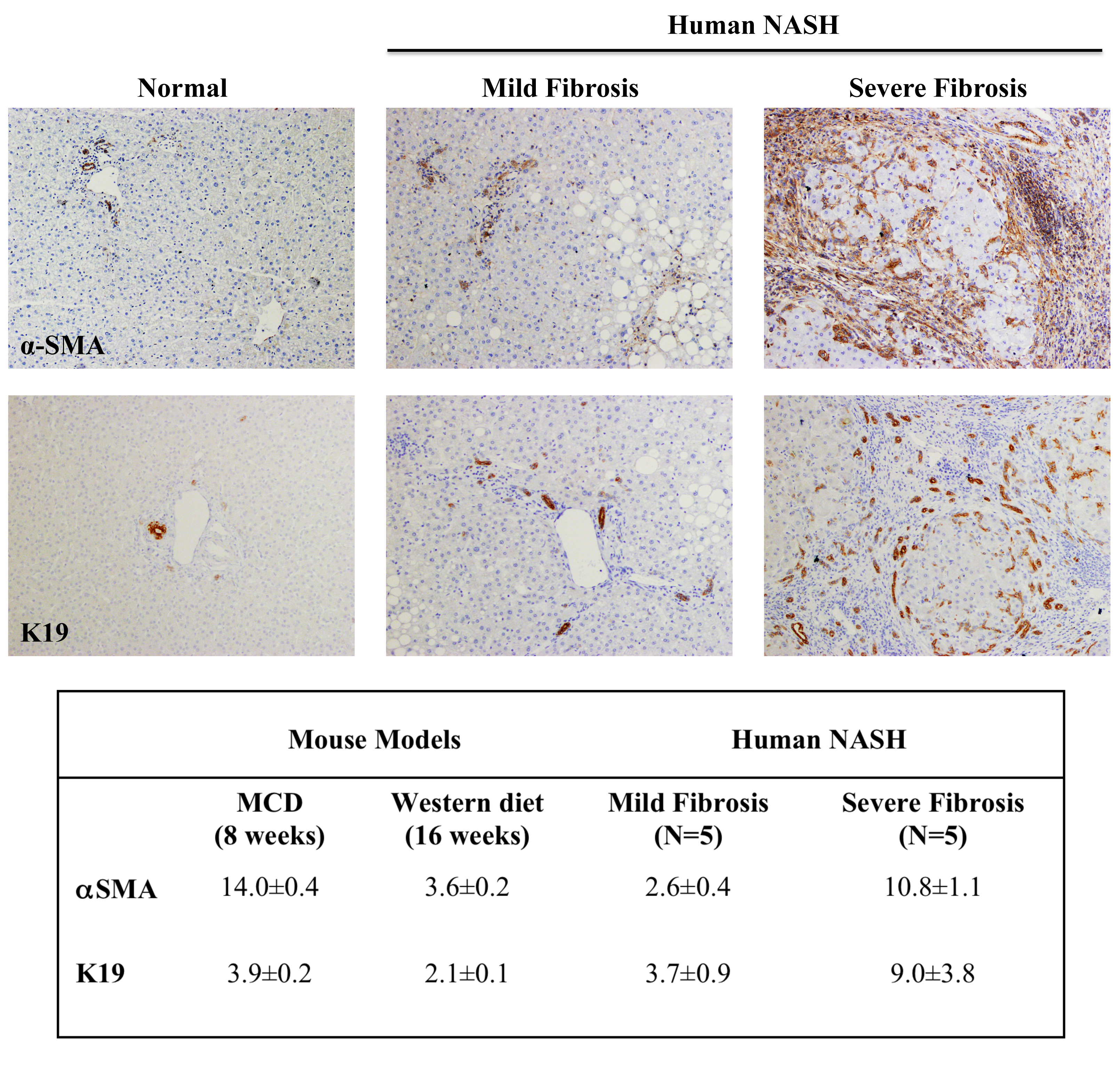

Supplement: S2 Fig — Representative photographs from immunohistochemistry for α-SMA and K19 in liver sections from healthy donors (N = 5), adult patients with NASH and mild fibrosis (N = 5) or severe fibrosis (N = 5). Table comparing morphometry for the above staining in murine models and human samples. Results are expressed in fold-change from chow diet (mouse model) or normal human liver (human NASH samples), with average±SEM. All results were statistically different from respective controls (P<0.05). (TIF) [file pone.0127991.s002.tif]

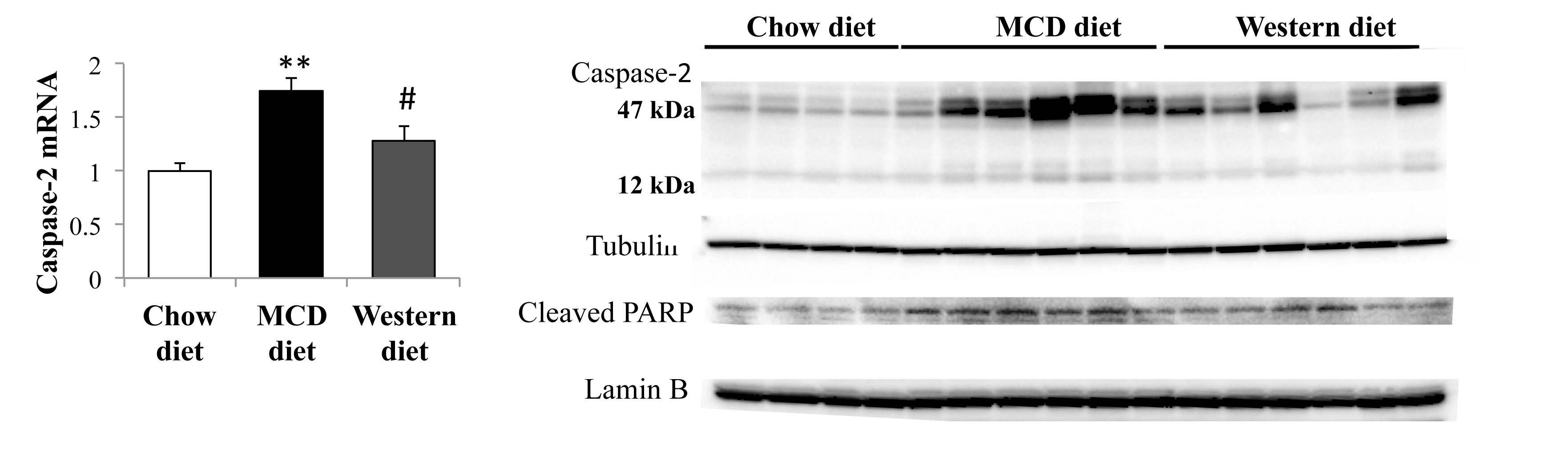

Supplement: S3 Fig — qRT-PCR analysis and Western blot for caspase-2 and cleaved PARP. Results normalized to chow-diet fed mice and graphed as mean±SEM. *<0.05 and **<0.005, control versus experimental diet; ##<0.005, MCD versus Western diet. (TIF) [file pone.0127991.s003.tif]

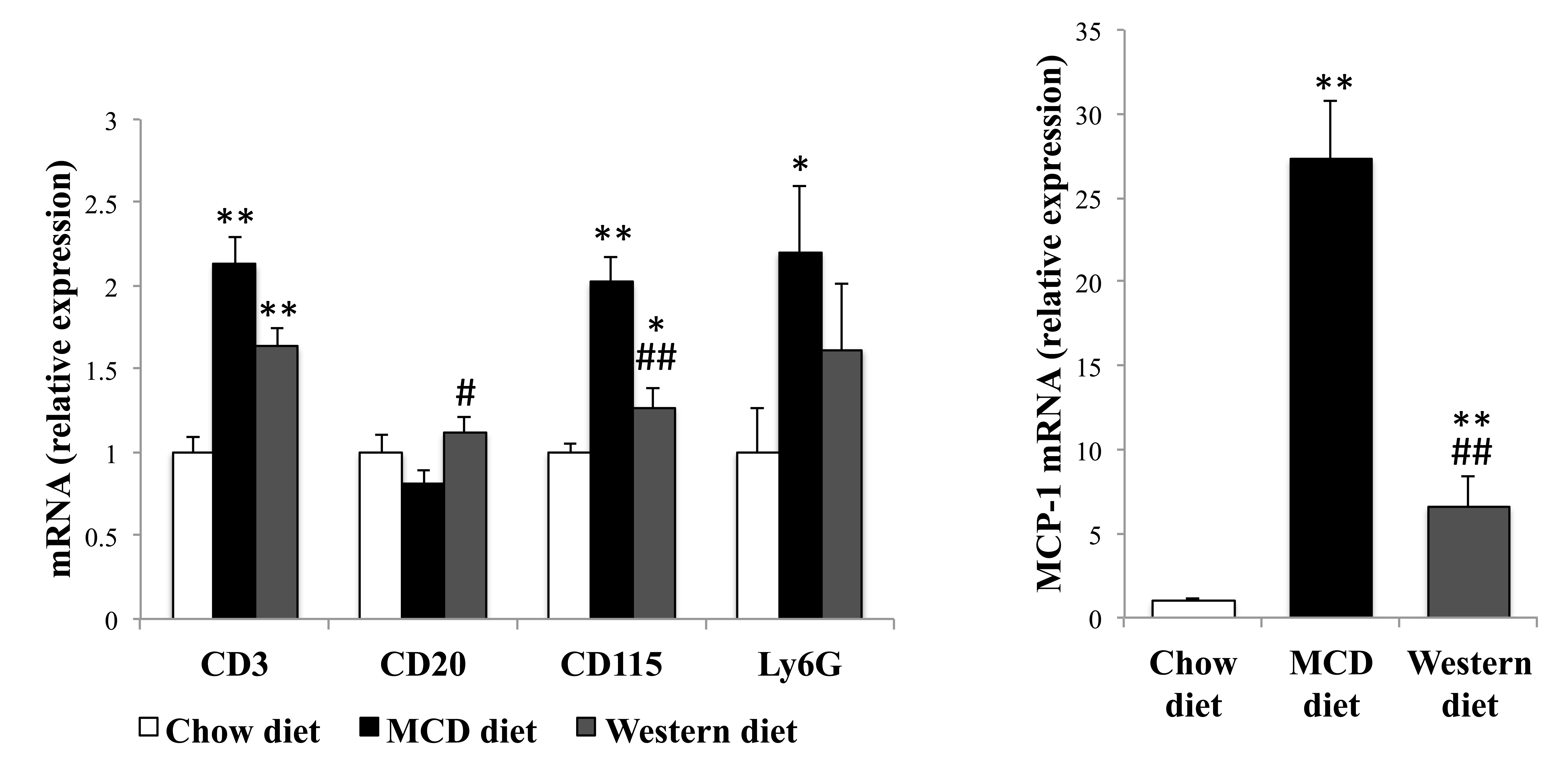

Supplement: S4 Fig — qRT-PCR analysis in whole liver, for: CD3 (pan-T lymphocyte marker), CD20 (pan-B lymphocyte marker), CD115 (marker of blood monocytes) and Ly6G (the granulocyte differentiation antigen 1). Results normalized to chow-diet fed mice and graphed as mean±SEM. *<0.05 and **<0.005, control versus experimental diet; ##<0.005, MCD versus Western diet. (TIF) [file pone.0127991.s004.tif]
