# Supplementary material for: Mouse Models of Diet-Induced Nonalcoholic Steatohepatitis Reproduce the Heterogeneity of the Human Disease
Source: PLoS One. 2015 May 27;10(5):e0127991. doi: 10.1371/journal.pone.0127991 (PMC4446215; doi:10.1371/journal.pone.0127991)
Supplement: S1 Table — (DOCX) [file pone.0127991.s005.docx]

**S1 Table. Characteristics of the diets**

|  | Chow diet | MCD diet | Western diet |
| --- | --- | --- | --- |
| Reference | Picolab Rodent diet 20, #5053 | MP Biomedicals, #960439 | TD.120330, 22% HVO + 0.2% cholesterol diet, Teklad Research |
| Kcal/g of diet | 4.7 | 4.5 | 4.6 |
| % Fat as calories | 13 | 20 | 45.3 |
| % CH as calories | 62 | 58 | 37 |
| % Proteins as calories | 24 | 22 | 17.7 |
