# Supplementary material for: Mouse Models of Diet-Induced Nonalcoholic Steatohepatitis Reproduce the Heterogeneity of the Human Disease
Source: PLoS One. 2015 May 27;10(5):e0127991. doi: 10.1371/journal.pone.0127991 (PMC4446215; doi:10.1371/journal.pone.0127991)
Supplement: S2 Table — (DOCX) [file pone.0127991.s006.docx]

**S2 Table. Composition of the experimental diets**

|  | MCD diet | Western diet^*^ |
| --- | --- | --- |
| Reference | MP Biomedicals, #960439 | TD.120330, 22% HVO + 0.2% cholesterol diet, Teklad Research |
| Formula (g/Kg) | Sucrose (455.3)  Corn Starch (200.0)  Corn Oil (100.0)  Alphacel Non-Nutritive Bulk (30.0)  Mineral Mix, AIN-76, (35.0)  Calcium Phosphate, dibasic (3.0)  L-Alanine (3.5)  L-Arginine HCl (12.1)  L-Asparagine Monohydrate (6.0)  L-Aspartic Acid (3.5)  L-Cystine (3.5)  L-Glutamic Acid (40.0)  Glycine (23.3)  L-Histidine HCl (4.5)  L-Isoleucine (8.2)  L-Leucine (11.1)  L-Lysine HCl (18.0)  L-Phenylalanine (7.5)  L-Proline (3.5)  L-Serine (3.5)  L-Threonine (8.2)  L-Tryptophan (1.8)  L-Tyrosine (5.0)  L-Valine (8.2)  Vitamin E, DL-alpha tocopherol acetate (250 IU/g) (0.484)  Vitamin A Palmitate (250,000 IU/g) (0.0792)  Vitamin D3, cholecalciferol (400,000 IU/g) (0.0055)  Ethoxyquin (0.02)  Biotin (0.0004)  Calcium Patothenate (0.0661)  Folic acid (0.002)  Inositol (0.1101)  Menadione (0.0496)  Niacin (0.0991)  p-Aminobenzoic acid (0.1101)  Pyridoxine HCl (0.0220)  Riboflacin (0.022)  Thiamine HCl (0.022)  Vitamin B12 (0.1% trit.) (0.0297)  Ascorbic acid (1.0166)  Corn Starch (3.4503) | Casein (230.0)  DL-Methionine (3.4)  Sucrose, fine ground (211.7098)  Corn Starch (80.0)  Maltodextrin (140.0)  Vegetable Shortening, hydrogenated (Primex) (220.0)  Soybean Oil (10.0)  Cholesterol (2.0)  Cellulose (50.0)  Mineral Mix, AIN-93G-MX (94046) (46.0)  Calcium Phosphate, dibasic (3.3)  Niacin (0.042)  Calcium Pantothenate (0.0224)  Pyridoxine HCl (0.0098)  Thiamin HCl (0.0084)  Riboflavin (0.0084)  Folic Acid (0.0028)  Biotin (0.0003)  Vitamin B12 (0.1% in mannitol) (0.035)  Vitamin E, DL-alpha tocopherol acetate (500 IU/g) (0.1)  Vitamin A Palmitate (500,000 IU/g) (0.0112)  Vitamin D3, cholecalciferol (500,000 IU/g) (0.0028)  Vitamin K1, phylloquinone (0.0011)  Choline Bitartrate (3.3)  TBHQ, antioxidant (0.046) |

*This diet is a modification of TD.06303 to add 0.2% cholesterol. We also add 42 g/L glucose and fructose in the drinking water (55% fructose and 45% glucose, w/w).
