# Supplementary material for: Mouse Models of Diet-Induced Nonalcoholic Steatohepatitis Reproduce the Heterogeneity of the Human Disease
Source: PLoS One. 2015 May 27;10(5):e0127991. doi: 10.1371/journal.pone.0127991 (PMC4446215; doi:10.1371/journal.pone.0127991)
Supplement: S3 Table — (DOCX) [file pone.0127991.s007.docx]

**S3 Table. Primaries Antibodies for Immunohistochemistry**

| **Antibody** | **Host** | **Company** | **Catalog #** | **Retrieval** | **Dilution** |
| --- | --- | --- | --- | --- | --- |
| α-SMA | Rabbit | Abcam | ab32575 | Citrate | 1:400 |
| Desmin | Rabbit | Abcam | Ab15200 | Citrate | 1:400 |
| K19 | Rat | Develpmental Studies Hybridoma Bank | Troma III | Pepsin + EDTA | 1:150 |
| Sox-9 | Rabbit | EMD Millipore | AB5535 | Citrate | 1:4000 |
| Osteopontin | Goat | R&D systems | AF808 | Pepsin | 5ug/mL |
| F4/80 | Rat | AbD Serotec | MCA497GA | Citrate | 1:150 |
| YM-1 | Rabbit | Stem Cell Technologies | #01404 | Citrate | 1:4000 |
| 4-Hydroxynonenal | Rabbit | Abcam | Ab46545 | Citrate | 1:250 |

α-SMA, alpha smooth muscle actin; K19, Keratin 19.
