# Supplementary material for: Mouse Models of Diet-Induced Nonalcoholic Steatohepatitis Reproduce the Heterogeneity of the Human Disease
Source: PLoS One. 2015 May 27;10(5):e0127991. doi: 10.1371/journal.pone.0127991 (PMC4446215; doi:10.1371/journal.pone.0127991)
Supplement: S4 Table — (DOCX) [file pone.0127991.s008.docx]

**S4 Table. RT-PCR primers for analysis**

| *Gene* | *Primer forward* | *Primer reverse* |
| --- | --- | --- |
| S9 | GACTCCGGAACAAACGTGAGGT | CTTCATCTTGCCCTCGTCCA |
| CPT-1a | TCCACCCTGAGGCATCTATT | ATGACCTCCTGGCATTCTCC |
| PPAR-α | AGAGCCCCATCTGTCCTCTC | ACTGGTAGTCTGCAAAACCAAA |
| ACOX | ATGCCTTTGTTGTCCCTATC | CCATCTTCAGGTAGCCATTATC |
| SCD-1 | CGTCTGGAGGAACATCATTC | AGCGCTGGTCATGTAGTA |
| MTTP | TCTCACAGTACCCGTTCTT | TCTTCTCCGAGAGACATATCC |
| ApoB | GGACTGTCTGACTTCCATATTC | AAGACTTGCCACCCAAAG |
| FAS | CTGCGGAAACTTCAGGAAATG | GGTTCGGAATGCTATCCAGG |
| ACC-α | AGGAGGACCGCATTTATCGAC | TGACCGTGGGCACAAAGTT |
| PPAR-γ | AGGCCGAGAAGGAGAAGCTGTTG | TGGCCACCTCTTTGCTCTGCTC |
| SREBP-1c | GCTACCGGTCTTCTATCAATG | GCAAGAAGCGGATGTAGTC |
| α-SMA | GTGGGGGACGAAGCGCAGAG | GGCCTTAGGGTTCAGCGGCG |
| Desmin | TACACCTGCGAGATTGATGC | ACATCCAAGGCCATCTTCAC |
| Col1α1 | TTCCCTGGACCTAAGGGTACT | TTGAGCTCCAGCTTCGCC |
| TGF-β | TTGCCCTCTACAACCAACACAA | GGCTTGCGACCCACGTAGTA |
| K-7 | TAGAGTCCAGCATCGCAGAG | CACAGGTCCCATTCCGTC |
| K-19 | GTGAAGATCCGCGACTGGT | AGGCGAGCATTGTCAATCTG |
| SOX-9 | CAGCAAGAACAAGCCACAACGTCAA | TTGTCCGTTCTTCACCGACTTCCT |
| α-Fetoprotein | TCGTATTCCAACAGGAGG | AGGCTTTTGCTTCACCAG |
| Caspase-2 | CAATGCTAACTGTCCAAGTCTA | GGGATTGTGTGTGGTTCTT |
| F4/80 | CAACACTCTCGGAAGCTATTAT | GAATTCCTGGAGCACTCATC |
| YM-1 | TCTGGTGAAGGAAATGCGTA | AATGATTCCTGCTCCTGTGG |
| TNF-α | TCGTAGCAAACCACCAAGTG | AGATAGCAAATCGGCTGACG |
| IL-6 | GACCTGTCTATACCACTTCAC | GTGCATCATCGTTGTTCATAC |
| Shh | CTGGCCAGATGTTTTCTGGT | GATGTCGGGGTTGTAATTGG |
| Ihh | CTCAGACCGTGACCGAAATAAG | TGGGCCTTGGACTCGTAATA |
| Patch | ATGCTCCTTTCCTCCTGAAACC | TGAACTGGGCAGCTATGAAGTC |
| Gli-1 | CCTCCTCCTCTCATTCCACA | CTCCCACAACAATTCCTGCT |
| Gli-2 | CCCCATCACCATTCATAAGC | CTGCTCCTGTGTCAGTCCAA |
| OPN | TGCACCCAGATCCTATAGCC | CTCCATCGTCATCATCATCG |
| Hip | AATTATGCCGCTACCTCTCG | CAGCATGTACCATTGCAACC |
| SOD-1 | GAGACCTGGGCAATGTGACT | GTTTACTGCGCAATCCCAAT |
| SOD-2 | CCGAGGAGAAGTACCACGAG | GCTTGATAGCCTCCAGCAAC |
| GSS | GCCTCCTACATCCTCATGGA | CCACATGCTTGTTCATCACC |
| GPx-1 | ACTACACCGAGATGAACGA | GACGTACTTGAGGGAATTCAG |
| Catalase | AGATGGAGAGGCAGTCTATT | AGATCTCGGAGGCCATAAT |
| Heme Oxigenase | GCTCGAATGAACACTCTGG | GTTCCTCTGTCAGCATCAC |
| GADD 153 | CTGGAAGCCTGGTATGAGGAT | CAGGGTCAAGAGTAGTGAAGGT |
| XBP-1 | TCCGCAGCACTCAGACTATG | ACAGGGTCCAACTTGTCCAG |
| GPR-78 | CGAAGGGATCATCTGCTATTAC | CTTCATAGTCCTGCCCATTG |
| ATF-4 | GCTATGGATGATGGCTTGG | CGAAACAGAGCATCGAAGT |
| ATF-6 | CGAAGGGATCATCTGCTATTAC | CTTCATAGTCCTGCCCATTG |
| UCP-1 | AGGCTTCCAGTACCATTAGGT | CTGAGTGAGGCAAAGCTGATTT |
| UCP-2 | ATGGTTGGTTTCAAGGCCACA | CGGTATCCAGAGGGAAAGTGAT |
| UCP-3 | CGAATTGGCCTCTACGA | TGTAGGCATCCATAGTCCC |
| CD3 | GATGCGGTGGAACACTTT | CTCTACACTGGTTCCTGAGA |
| CD20 | TCTACGACTGTGAACCATCTA | GTTTCTGGAAGAAGGCAGAG |
| CD115 | CTGTATGTCTGTCATGTCTCTG | GCTATTGCCTTCGTATCTCTC |
| Ly6G | TTCCTGCAACACAACTACC | GATGGGAAGGCAGAGATTG |
| MCP-1 | AGGTCCCTGTCATGCTTCTG | TCTGGACCCATTCCTTCTTG |

CPT1a, Carnitine Palmitoyltransferase 1A; PPAR-α, Peroxisome Proliferator-Activated Receptor-α; ACOX, Acyl Coenzyme A Oxidase; SCD-1, Stearoyl-CoA Desaturase; MTTP, Microsomal Triglyceride Transfer Protein; ApoB, Apolipoprotein B; FAS, Fatty Acid Synthase; ACC-α, Acetyl-CoA Carboxylase-α; PPAR-γ, Peroxisome Proliferator-Activated Receptor-γ; SREBP-1c, Sterol Regulatory Element Binding Protein-1c; α-SMA, α-smooth muscle actin; Col1α1, Collagen-α1; TGF-β, Transforming Growth Factor-β; K7, Keratin 7; K19, Keratin 19; TNF-α, Tumor Necrosis Factor-α; IL-6, Interleukin-6; Shh, Sonic Hedgehog; Ihh, Indian Hedgehog; OPN, Osteopontin; Hip, Hedgehog Interacting Protein; SOD-1 and -2, Superoxide Dismutase-1 and-2; GSS, Glutathione Synthetase; GPx1, Glutathione Peroxidase; XBP-1, ; GPR-78, ;ATF-4 and -6, Activating Transcription Factor-4 and -6; Ly6G, Lymphocyte Antigen 6G; MCP-1, Monocyte Chemoattractant Protein-1.
