# Supplementary material for: Mouse Models of Diet-Induced Nonalcoholic Steatohepatitis Reproduce the Heterogeneity of the Human Disease
Source: PLoS One. 2015 May 27;10(5):e0127991. doi: 10.1371/journal.pone.0127991 (PMC4446215; doi:10.1371/journal.pone.0127991)
Supplement: S5 Table — (DOCX) [file pone.0127991.s009.docx]

**S5 Table. Primary Antibodies for Western Blot**

| **Antibody** | **Host** | **Company** | **Catalog Number** | **Dilution** |
| --- | --- | --- | --- | --- |
| Gli-2 | Rabbit | Genway | GWB-CE7858 | 1:500 |
| Caspase-2 | Mouse | Cell Signaling Technology | #2224 | 1:500 |
| Cleaved PARP | Rabbit | Cell Signaling Technology | #9544 | 1:500 |
| GADD 153 | Rabbit | Abcam | Ab27539 | 1:500 |
| LC3 Antibody | Rabbit | Cell Signaling Technology | #2775 | 1:500 |
| α-Tubulin | Rabbit | Abcam | Ab4074 | 1:5000 |
| Lamin B1 | Rabbit | Abcam | AB16048 | 1:1000 |

LC3, microtubule-associated protein 1A/1b-light chain 3.
